# The Effect of 3D Nanofibrous Scaffolds on the Chondrogenesis of Induced Pluripotent Stem Cells and Their Application in Restoration of Cartilage Defects

**DOI:** 10.1371/journal.pone.0111566

**Published:** 2014-11-12

**Authors:** Ji Liu, Huarong Nie, Zhengliang Xu, Xin Niu, Shangchun Guo, Junhui Yin, Fei Guo, Gang Li, Yang Wang, Changqing Zhang

**Affiliations:** 1 Department of Orthopaedic Surgery, Shanghai Sixth People's Hospital, Shanghai Jiaotong University School of Medicine, Shanghai, China; 2 Institute of Polymers, School of Materials Science and Engineering, Nanchang University, Nanchang, China; 3 Department of Orthopaedics & Traumatology, Stem Cells and Regeneration Program, School of Biomedical Sciences, Room 904, Li Ka Shing Institute of Health Sciences, The Chinese University of Hong Kong, Prince of Wales Hospital, Shatin, Hong Kong, SAR, PR China; Instituto de Engenharia Biomédica, University of Porto, Portugal

## Abstract

The discovery of induced pluripotent stem cells (iPSCs) rendered the reprogramming of terminally differentiated cells to primary stem cells with pluripotency possible and provided potential for the regeneration and restoration of cartilage defect. Chondrogenic differentiation of iPSCs is crucial for their application in cartilage tissue engineering. In this study we investigated the effect of 3D nanofibrous scaffolds on the chondrogenesis of iPSCs and articular cartilage defect restoration. Super-hydrophilic and durable mechanic polycaprolactone (PCL)/gelatin scaffolds were fabricated using two separate electrospinning processes. The morphological structure and mechanical properties of the scaffolds were characterized. The chondrogenesis of the iPSCs *in vitro* and the restoration of the cartilage defect was investigated using scanning electron microscopy (SEM), the Cell Counting Kit-8 (CCK-8), histological observation, RT-qPCR, and western blot analysis. iPSCs on the scaffolds expressed higher levels of chondrogenic markers than the control group. In an animal model, cartilage defects implanted with the scaffold-cell complex exhibited an enhanced gross appearance and histological improvements, higher cartilage-specific gene expression and protein levels, as well as subchondral bone regeneration. Therefore, we showed scaffolds with a 3D nanofibrous structure enhanced the chondrogenesis of iPSCs and that iPSC-containing scaffolds improved the restoration of cartilage defects to a greater degree than did scaffolds alone *in vivo*.

## Introduction

Cartilage loss and the healing of arthritis is difficult and an optimal solution remains unavailable due to the poor blood supply and regenerative capability of cartilage. Recently, tissue engineering has become a promising treatment alternative. However, the availability of chondrogenic cells for cartilage tissue engineering is limited. Mesenchymal stem cells (MSCs) can be isolated from bone marrow, synovium, periosteum, skeletal muscle, and adipose tissue, and have been widely used for osteochondral repair. However, the invasive harvesting procedure, and difficulties in retaining the stemness and a prolonged proliferation capability has restricted their further application [1], [2]. Recently, interest in pluripotent or primitive stem cells has increased. Human embryonic stem cells (hESCs) can be differentiated into chondrocytes using certain procedures and conditions, but concerns regarding possible immunorejection as well as limited availability and ethical issues have been raised [3], [4]. Since Yamanaka and colleagues retrodifferentiated somatic cells to an ESC-like state, namely iPSCs, numerous reports regarding safer and more efficient methods for generating iPSCs for clinical applications have been published [5]–[7]. iPSCs can also be induced into a variety of cell lineages, including the osteochondral lineage [8], [9], and studies using scaffolds or gel carriers to enhance the chondrogenesis of iPSCs have been performed. A competent scaffold for cartilage reconstruction should provide the necessary mechanical strength, directed and controlled degradation, as well as the appropriate porosity to allow the nutrients and waste to diffuse, promoting cell proliferation [10], [11]. Rigid scaffolds, such as poly(lactic-co- glycolic acid; PLGA) and PLA can provide more support under load, which may be particularly important after the initial surgery, but may affect the properties of maturing chondrocytes and hyaline cartilage if not broken down appropriately; moreover, their application requires more invasive surgical implantation procedures [12], [13]. An alternative is natural or synthetic hydrogels [14], [15]. The advantages of gel scaffolds are that cells can be distributed homogenously before the polymerization process, and the scaffolds are highly permeable. However, gel scaffolds generally lack sufficient mechanical strength; thus, maintaining the original spatial structure and original site of implantation is difficult.

The electrospinning method produces continuous, randomly aligned polymeric nanofibers with diameters ranging from dozens of nanometers to several microns, although such scaffolds have considerable porosity and surface area as in a 3D structure [16]–[18]. Specific natural ingredients such as gelatin, glycosaminoglycan, chitosan and hyaluronic acid can promote the hydrophilicity and cell affinity of the scaffold [19], [20]. These features enable electrospun nanofibrous scaffolds to mimic the extracellular matrix (ECM) of cartilage.

Thus, we synthesized a nanofibrous scaffold using PCL and gelatin (ratio 50∶50) and investigated its effect on the chondrogenic induction of iPSCs, *via* embryoid body (EB) formation and high-cell-density culture *in vitro*; we implanted the cell-scaffold complex into an animal model of articular cartilage defect and assessed the efficacy of cartilage restoration *in vivo*.

## Materials and Methods

### 1. Scaffold preparation

All materials and reagents were purchased from Sigma-Aldrich (St. Louis, MO, USA) unless otherwise specified. PCL/gelatin nanofibrous scaffolds were fabricated by electrospinning using two separate cylinder and power controllers: PCL (MW, 70–90 kD) was dissolved (10%, w/v) in an emulsion solution containing dichloromethane, dimethylbenzene and Span 20 at a volume ratio of 5/3/0.05. Gelatin (type-I gelatin from bovine skin, MW 40–50 kD) at a weight ratio of 20% (w/v) was also prepared in a 2∶1 (v/v) formic acid: ethyl ester mixture. The electrospinning process was conducted at 30°C and the collecting distance was fixed at 25 cm. For the blend electrospinning process, the collecting drum (rotation speed, 500 rpm) was connected to a negative power supply (−6 kV). The applied positive voltages for PCL emulsion and gelatin solutions were 24 and 27 kV, respectively. The process was conducted for 2 h with feeding rates of 1 mL/h for PCL emulsion and 0.5 mL/h for gelatin solution. Fibrous mats of the required size were dried under vacuum to evaporate residual organic solvent.

### 2. Scaffold characterization

#### 2.1 Scanning electron microscopy (SEM) observation and measurements

The scaffold morphologies were observed using a scanning electron microscope (SEM, HITACHI S-4800, Japan) at an accelerating voltage of 10 kV. Each sample was sputter-coated with platinum for analysis. The mean fiber diameter and porosity were calculated using the Image J 1.40 g software.

#### 2.2 Mechanical characterization

The cut scaffolds 3×1 cm in size were tested in terms of their mechanical properties, including stress-strain curve, stress and strain at break, stress at yield, and Young's modulus, using a tensile testing machine (Model 5948 Micro-Tester, Instron Co., Norwood, MA, USA) according to the manufacturer's protocol.

#### 2.3 *In vitro* scaffold degradation

*In vitro* degradation was evaluated by determining the weight loss and evaluating the surface morphology of the scaffolds (n = 3). The scaffolds (3×1 cm) were immersed in 10-mL 4% PBS (pH = 7.4) solution at 37°C for 2 months. The PBS was changed every 7 days and the scaffolds were dried and weighed. The percent degradation for each sample was calculated by dividing the weight loss by the initial dry weight, and the final scaffolds were examined in terms of their surface morphology and mechanical characteristics.

### 3 *In vitro* chondrogenesis of iPSCs on the scaffolds

#### 3.1 *In vitro* culture of iPSCs and formation of EBs

Mouse iPSCs (S103F9) derived from mouse dermal fibroblasts were kindly provided by Professor Pei [21]. The iPSCs were routinely cultured on a feeder layer of mitomycin-inactivated mouse fibroblasts in a cultivation medium consisting of Dulbecco's modified Eagle's medium (DMEM; Gibco, Invitrogen, Grand Island, NY, USA) supplemented with 15% fetal bovine serum (FBS; SAFC Biosciences, Lenexa, KS, USA), 2 mmol/L L-glutamine (Gibco, Invitrogen), 0.4 mL β-mercaptoethanol (Sigma-Aldrich) and nonessential amino acids (Gibco, Invitrogen). For formation of EBs, the cells were trypsinized, counted and adjusted to 10^5^ cells/mL. Next, 25- µL drops (2−5×10^3^ cells per drop) of medium were placed onto the inside surface of the dish lid by serial pipetting. After 2 days of culture, each drop with one EB suspended in the center was evaluated, collected, and cultured in a 10-cm gelatin-coated dish.

#### 3.2 *In vitro* cell proliferation assay

Before further procedures, the scaffolds were sterilized on both sides with UV light for 2 h and cut into smaller pieces (1×1 cm). Scaffold biocompatibility and cytotoxicity were analyzed using the CCK-8 kit (Dojindo Laboratories, Kumamoto, Japan). Each well was filled with 0.5-mL medium; 50- µL of CCK-8 solution was then added at 3 h and 1, 3, 7 and 14 days. Next, the cells were incubated at 37°C for 2 h. The medium in the wells was extracted for absorbance measurement at 450 nm using a microplate reader (Bio-Rad, Berkeley, CA, USA). Three wells per group were subjected to replicate testing at each time point.

#### 3.3 Culturing and chondrogenesis of iPSCs on the scaffolds

For chondrogenesis, the EBs were cultured for 5 days, trypsinized into single cells and counted. Next, three drops of 15- µL medium each containing 3×10^5^ cells were pipetted onto the center of the scaffolds, which were placed in a 24-well plate. The seeded cells were allowed to attach for 2 h, and then each well was supplemented with 0.5-mL chondrogenesis differentiation medium (Invitrogen) containing high-glucose DMEM with 10% FBS, 6.25 µg/mL insulin, 6.25 µg/mL transferrin, 50 µmol/mL ascorbic acid, 100 nmol/L dexamethasone and 10 ng/mL TGF-β1, according to the manufacturer's instructions. Identical numbers of of cells were cultured directly in the wells as a control. The medium was changed every 2 days and the cells were collected at 2 and 3 weeks for further analysis.

#### 3.4 SEM

The attachment of cells to the scaffolds was observed using SEM. Scaffolds with attached cells were rinsed three times with PBS, fixed in 2.5% glutaraldehyde at 4°C for 1 h, dehydrated through increasing concentrations of ethanol, and critical point-dried, gold sputter-coated, and observed using a SEM (HITACHI S-4800).

#### 3.5 Immunofluorescence

Immunohistochemical staining was used to detect the ECM produced by the chondrogenically induced cells on the scaffolds. Briefly, scaffolds with cells were rinsed and fixed as described above, and blocked with 1% bovine serum albumin in PBS for 1 h. Then, the samples were incubated with anti-collagen II antibody (mouse clone, 1∶50; Millipore) or anti-aggrecan antibody (rabbit clone, 1∶50; Millipore) at 4°C overnight, rinsed with PBS and incubated with an Alexa Fluor 555 anti-mouse antibody (goat clone, 1∶800; Invitrogen) at 37°C for 30 min. The samples were mounted with mounting medium containing DAPI (Vector, Burlingame, CA, USA) and observed under a Leica DM 3000 fluorescence microscope.

#### 3.6 Quantitative real-time polymerase chain reaction (qRT-PCR)

Total RNA was extracted from the differentiated iPSCs using TRIZOL reagent (Invitrogen) according to the manufacturer's instructions. After reverse transcription, quantitative real-time polymerase chain reaction (qRT-PCR) was performed using a TP800 system (Takara, Japan) with SYBR Premix Ex Taq (Takara). The qRT-PCR conditions were 40 cycles at 95°C for 10 s, 60°C for 20 s and 72°C for 20 s. GAPDH was used as the internal control. The primer sequences used are listed in Table 1. Relative mRNA expression of cells cultured on scaffolds and plates was calculated.

**Table 1:** Real-time PCR primers and product sizes.

Gene	Sequence(5′-3′)		Size(bp)
Col-II	sense	GGACGCTCAGGAGAAACAGG	221
	antisense	TTGTTCAGTGACTTGAGTGTAGCG	
Col-I	sense	AGGGTGAGACAGGCGAACA	184
	antisense	CCGTTGAGTCCGTCTTTGC	
Aggrecan	sense	GATCGACCAGAAGCTGTGTGAG	250
	antisense	TTGCAAGGAGTGTCCATCTGA	
Sox9	sense	GAAAGACCACCCCGATTACAAG	236
	antisense	CGTCGGTTTTGGGAGTGGT	
GAPDH	sense	GCATGGGCCAGAAGGACTCGTA	214
	antisense	TCGCGGTTGGCCTTGGGGTTCA	

#### 3.7 Western blotting

For western blot analysis, cells were lysed in mammalian protein extraction reagent (Pierce, Rockford, IL, USA) with complete protease inhibitor. Total protein quantity was determined using the Pierce BCA Protein Assay Kit (Pierce) and adjusted to 10 mg/mL. Samples (10 mL) were loaded onto 10–14% SDS-PAGE gels and transferred to a polyvinylidene fluoride membrane (Millipore, Billerica, MA, USA). The membranes were then blocked with 5% skim milk in 0.05% Tris-buffered saline/Tween 20 (TBST) followed by incubation with the following primary antibodies: anti-sox9 (Abcam, Cambridge, England, 1∶1000), anti-aggrecan (Abcam, 1∶100), anti-collagen type I (Merck, Whitehouse Station, NJ, USA, 1∶500), anti-collagen type II (Merck, 1∶500) and anti-GAPDH (Cell Signaling, Danvers, MA, USA, 1∶1000), respectively. The blots were developed for chemiluminescence using the ECL western blotting substrate (Pierce).

### 4. Investigation of scaffold-cell complexes *in vivo*

#### 4.1 Animal surgery

Thirty-nine mature New Zealand rabbits (2.5–3.0 kg) were used in the experiment. All procedures and handling of the animals were approved by the Animal Research Committee of Shanghai Jiaotong University School of Medicine and surgeries were performed under isoflurane anesthesia, with every effort made to minimize the animals' suffering. Seventy-two knees of 36 rabbits were used to establish the cartilage defect model. Briefly, after the animals had been anesthetized with an intravenous injection of pentobarbital, a lateral parapatellar skin incision was made. The knee joint was exposed when the joint capsule was sliced open and the patella was extracted laterally. Under direct vision, a full-thickness cartilage and subchondral bone defect 5 mm in diameter and 3 mm in depth was made using a 5-mm sterile stainless-steel drill. Then, the rabbits were randomly divided evenly into three groups: the cell group, with the defects covered by the iPSC-seeded scaffolds (10^5^ cells per scaffold, derived from EBs cultured for 5 days), which were resized before the surgery; the scaffold group, with the defects covered by cell-free scaffolds; and the control group, with the defects uncovered. The remaining three rabbits underwent the same procedure except that no cartilage defect was made, and were used as positive controls for micro-CT analysis and as a reference in histological scoring assessments. All incisions were sutured and each rabbit was given 8 million IU of penicillin postoperatively and placed back in their cage. To suppress the immunorejection caused by heterogeneous cell transplantation, 10 mg/kg cyclosporine was injected subcutaneously every 2 days postoperatively for 2 months. The rabbits were allowed to move freely. In each group, six rabbits were sacrificed at the end of the first 6 weeks and six at the end of month 3 with an overdose of anesthetic. The three positive control rabbits were also sacrificed. Samples of the distal femur were collected and treated as described below for analysis.

#### 4.2 Gross and histological evaluations

The femoral condyle samples were dissected several millimeters above the boundary of cartilage coverage and fixed in 4% paraformaldehyde for 72 h and photographed. Next, the fixed femoral condyles were decalcified in 10% EDTA for 1 month at room temperature. The samples were then grade-dehydrated with ethanol and embedded in paraffin. Sections were cut in 4-mm sagittal slices and stained with hematoxylin and eosin (H & E) for general histological observation as well as safranin-O for assessment of cartilaginous ECM composition.

#### 4.3 qRT-PCR and western blotting

Three months postoperatively three rabbits per group were euthanized and the tissue inside the defect area, which was marked using an annular drill, was scraped off with a scalpel and collected. The cartilage fragments were immediately frozen and ground with a mortar and pestle. Total RNA was extracted with TRIZOL (Invitrogen) for qRT-PCR analysis of collagen I, collagen II, aggrecan and sox9 gene expression. For western blotting, the tissue was homogenized with tissue protein extraction reagent (Pierce) according to the manufacturer's instructions, and the levels of the four proteins were determined. β-actin and GAPDH were used as controls for qRT-PCR and western blotting, respectively.

#### 4.4 Micro-CT scanning

Three months postoperatively, micro-CT scanning (Skyscan1172, Bruker-microct, Belgium) was utilized to visualize the microstructural morphology of the subchondral bone reconstruction in the defect area. After fixation in 4% formaldehyde for 3 days, the samples were immobilized with the femoral axis perpendicular to the scanning plane. The entire femoral condyle was scanned and the original defect area, measuring 5 mm in diameter and 1 mm in depth, was selected as the region of interest (ROI). The 3D image was established and the quality of the regenerated bone in the ROI was assessed and presented as the average bone mineral density (BMD).

### 5 Statistical analyses

The quantitative data were obtained from triplicate experiments and presented as means ± standard deviation (SD). Statistical significance was analyzed using ANOVA or Student's *t*-test. A value of p<0.05 was taken to indicate statistical significance.

## Results

### 1. Scaffold characterization

#### 1.1 Morphology and measurements

**Figure 1:**
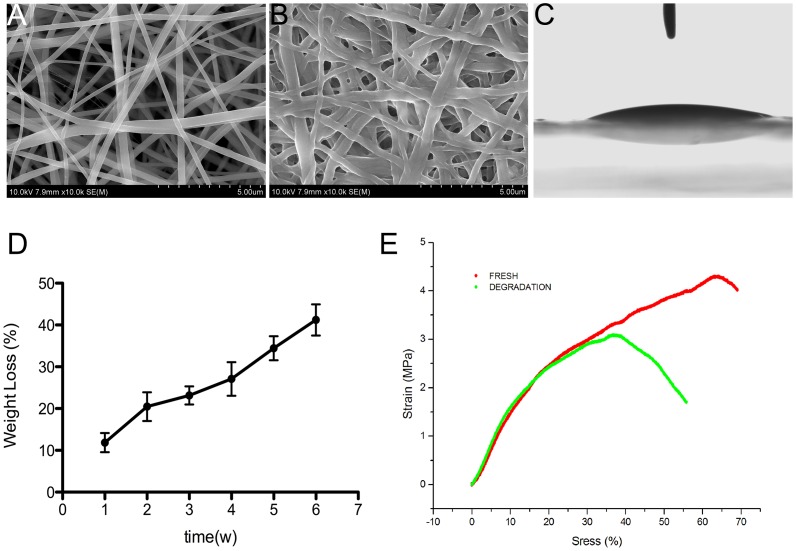
Scaffold characterization. (**A**) SEM images of the scaffold revealed a mesh-like nanofibrous structure. (**B**) SEM image of a scaffold degraded in PBS solution for 1 month. (**C**) Image of water droplet on the scaffold. (**D**) Scaffold weight loss during degradation. (**E**) Elastic curves of intact and 1-month degraded scaffolds in red and green, respectively.

As shown in Figure 1(A), the PCL and gelatin-composite scaffolds exhibited randomly oriented non-woven fibers with an open porosity and interconnected pores. The mean fiber diameter was 305±72 nm. The scaffold thickness was 50±5.56 µm and the porosity, 96%.

Providing tissue engineering scaffolds with superior hydrophilicity is important due to enhance cellular attachment and proliferation. According to Laleh Ghasemi-Mobarakeh *et al*. [22], the water contact angle of PCL-alone scaffolds is 118°±6°, indicating that nanofibrous scaffolds with PCL only are hydrophobic and not suitable for cell attachment. Figure 1(C) shows representative water drop images (water contact angle, 16.25±1.37^o^) of the PCL/gelatin scaffolds, which confirmed that the presence of gelatin significantly improved their hydrophilicity. Furthermore, unlike the PCL fibrous membranes obtained from the solution, the prepared PCL scaffolds generated from the emulsion were also hydrophilic due to the amphiphilic properties of the remnant surfactant.

#### 1.2 Mechanical tests and *in vitro* degradation

Typical stress–strain curves before and after degradation and detailed mechanical properties of the scaffolds are presented in Figure 1(E) and Table 2, respectively. Since gelatin is water soluble, only the PCL component of the PCL–gelatin composite scaffold remained in the fibers after degradation in PBS buffer. According to the stress-strain curves, despite the gelatin dissolution, curves were similarly steep, indicating that PCL was the primary contributor to mechanical support in this scaffold blend. Compared to intact scaffolds, those after degradation showed lower elastic strength and strain at break but a similar modulus (Table 2). Gelatin dissolution also reduced scaffold elongation and elasticity.

**Table 2:** The mechanical properties of the scaffold.

Scaffold	Ultimate tensile stress(MPa)	Ultimate tensile strain(%)	Young's modulus(MPa)
Intact	4.31±0.31	63.01±12.63	5.63±0.22
Degraded	3.08±0.26	35.98±8.66	5.78±0.41

The weight of scaffolds immersed in PBS decreased gradually. Indeed, >20% of the weight loss occurred in the first 2 weeks. After 6 weeks of degradation, scaffold weight had decreased by almost 50%, indicating that the majority of the gelatin (50% of the initial weight) had diffused into the PBS buffer (Fig. 1D). Although slight curling and mutual merging were evident (Fig. 1B), the SEM image showed no obvious fracture of the PCL fibers after 2 months of degradation, confirming that no significant hydrolysis of PCL fibers occurred. As a result, although the ultimate tensile stress and strain of the degraded fibers were ∼40% lower than those of intact fibers, the Young's modulus was unchanged.

### 2 Cultivation and chondrogenesis of iPSCs

#### 2.1 EB formation and cultivation

**Figure 2:**
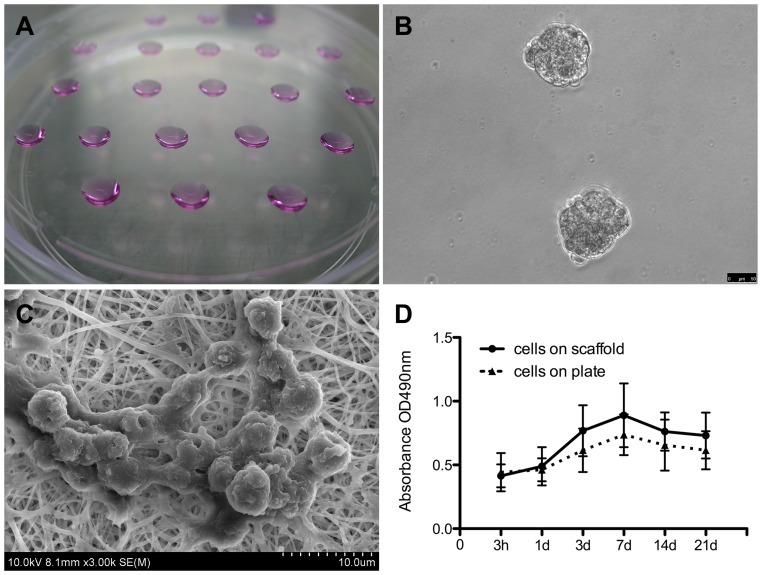
EB formation and the iPSC culture on the scaffold *in vitro*. (**A**) Formation of EBs *via* serial dripping. Each drop of culture medium contained ∼2−5×10^3^ cells, which congregated and developed into an EB (**B**). (**C**) A cluster of iPSCs on the scaffold. (**D**) Proliferation of the iPSCs cultured on the scaffolds and on the plate was determined using CCK-8 tests; results revealed that the scaffolds promoted cell growth.

Spherical, uniformly sized EBs were harvested using the serial pipetting method after suspension-culture of identical numbers of cells for 2 days. A further 5 days of cultivation resulted in enlargement and maturation of EBs for the following differentiation (Fig. 2A, B).

#### 2.2 iPSC morphology and proliferation

SEM images showed that most cells were attached to the surface of the scaffold and a portion infiltrated between the fibers with increasing culture duration (Fig. 2C). Furthermore, CCK-8 analysis confirmed cell proliferation and the cellular compatibility of the scaffold. The OD values were not significantly different between the two groups at each time point (Fig. 2D).

#### 2.3 iPSC chondrogenesis *in vitro*

**Figure 3:**
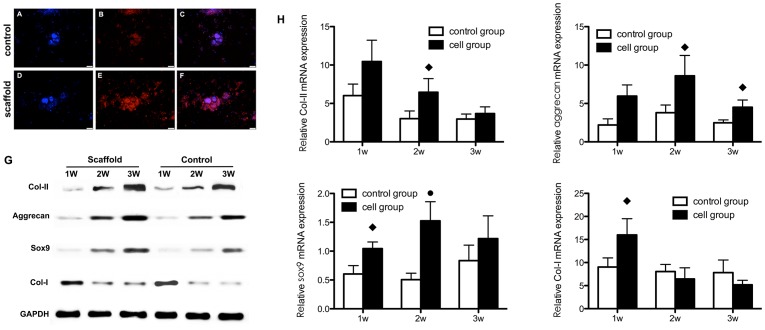
Chondrogenesis of iPSCs cultured on scaffolds (scaffold group) or plates (control group) *in vitro*. Collagen II synthesis in the scaffold group (**A,B,C**) and control group (**D,E,F**) was analyzed by immunofluorescence staining, including nuclear counterstaining with DAPI (**A,D**), anti-collagen II staining in red (**B,E**); merged images are also shown (**C,F**). (**G**) qRT-PCR analysis of gene expression and (**H**) western blotting for collagen II, aggrecan, sox9 and collagen I, using GAPDH as the internal reference. The nanofibrous scaffolds generally enhanced cartilage-specific gene expression and protein levels. ♦ Statistically significant difference compared with the control group (*p*<0.05); • Statistically significant difference compared with the control group (*p*<0.01).

The PCR analysis showed that the expression levels of chondrogenic marker genes (collagen type II, aggrecan and sox9) were elevated in both groups; moreover, expression levels were significantly higher in the scaffold group than the control group. Collagen type I expression did not change significantly in either group but was lower at the end of cultivation in the scaffold group (Fig. 3H). Western blotting analysis showed that the levels of the three chondrogenic markers were higher in the scaffold group than the control group. The collagen type I level in the scaffold group decreased with increasing culture duration (Fig. 3G).

The immunofluorescence analysis yielded similar results. The chondrogenic ECM synthesized and secreted by the chondrogenically differentiated iPSCs formed a mass around the cell nuclei (DAPI counterstained). The fluorescence intensity was greater in the scaffold group compared with the control group (Fig. 3A).

### 3 *In vivo* assessment of scaffolds and cells

#### 3.1 Macroscopic observation

All rabbits survived until sacrificed for sample harvesting. Parts of the knee joint appeared slightly red and swollen with an increased surface temperature in the first week after scaffold implantation. Six weeks postoperatively, defects covered with the scaffold-cell complex (cell group) were filled with grayish-white tissue. The surface of the regenerative tissue was rough and gaps were observed between the edges and normal cartilaginous tissue. Three months postoperatively, the regenerative tissue filled the defect almost entirely and the surface was glossy and level with the normal articular surface. Defects without scaffolds (blank group) changed minimally in the first 6 weeks, during which time the subchondral bone was exposed. In the following 6 weeks, coverage of the regenerative tissue increased, reaching the defect margin; however, the surface was rugged and fissures were present. The changes in defects filled with the cell-free scaffolds (control group), were intermediate between those of the other two groups. However, the regenerative tissue was less glossy and smooth compared to that in the cell group.

#### 3.2 Histological observation

**Figure 4:**
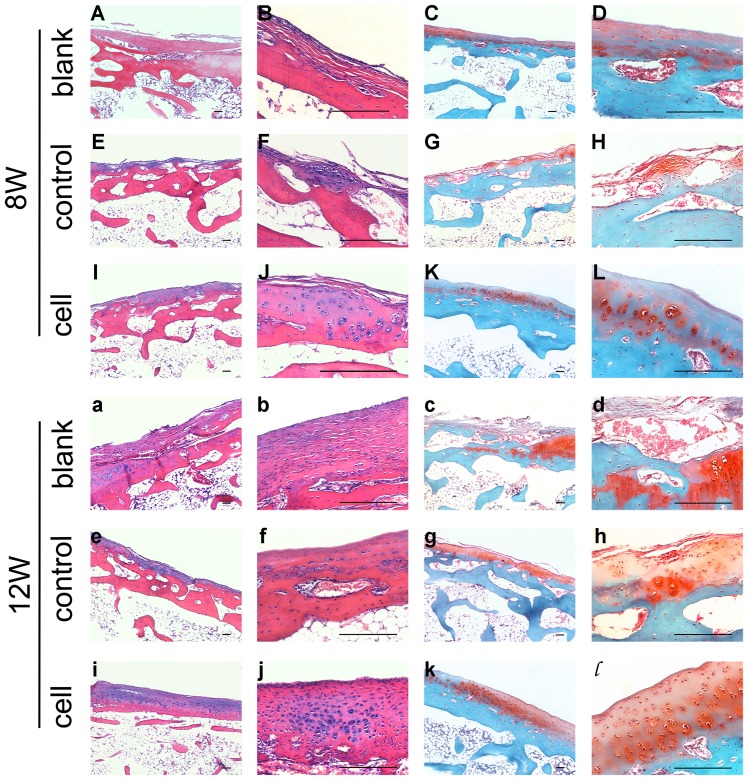
Histological evaluation of scaffolds and iPSCs in the restoration of cartilage defects at 8 weeks (A–L) and 12 weeks (a–l). Blank group (**A–D, a–d**) defects were filled with fibrocartilage, while abundant cells were observed in the cell group (**I–L, i–l**). The H&E and safranin-O stained images in the right column are magnifications of images in the left column. All scale bars represent 200 µm.

**Figure 5:**
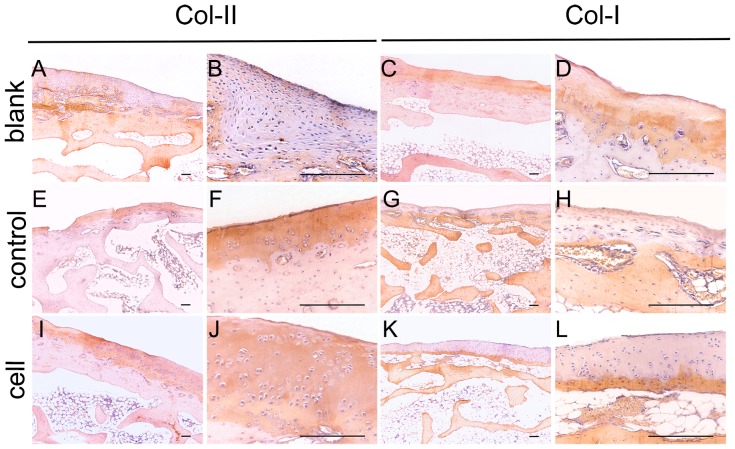
Immunohistochemical assessment of scaffolds and iPSCs in the restoration of cartilage defects at 12 weeks. More collagen II was observed in the cell and control groups, while more collagen I was observed in the blank group. All scale bars represent 200 µm.

Six weeks postoperatively, the defects in the blank and scaffold groups were covered only barely, and the subchondral bone was exposed. Three months later, fibrous tissue covering the subchondral bone was observed, which was stained less intensely than the normal cartilaginous tissue, and exhibited a streak texture instead of the homogeneous appearance of cartilage matrix. Safranin-O staining showed deposition of a small amount of cartilaginous matrix in the defect area. In the cell group, stronger staining of regenerated cartilaginous matrix was observed around the clusters of regenerated chondrocytes, especially at the end of the third month; immunohistological analysis showed identical results. Collagen II production was increased in the defect area in the cell group, while the collagen I content was lower. The changes in the cartilaginous matrix described above were also observed in the other two groups, and particularly in the blank group (Figs. 4, 5).

#### 3.3 qRT-PCR and western blot analysis

**Figure 6:**
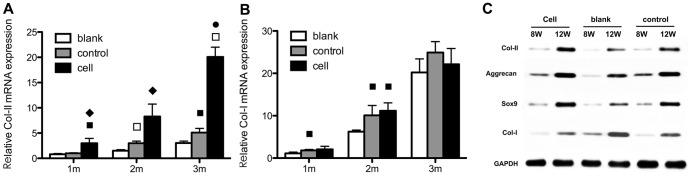
Gene expression and protein levels *in vivo*. Collagen I (**A**) and collagen II (**B**) gene expression levels over 3 months were determined using qRT-PCR, and western blotting was used to evaluate the collagen II, aggrecan, sox9 and collagen I protein levels in the regeneration area (**C**). The scaffold notably stimulated the expression of the cartilage-related markers, but not collagen I. ♦ Statistically significant difference compared with the control group (*p*<0.05); • Statistically significant difference compared with the control group (*p*<0.01); ^▪^ Statistically significant difference compared with the blank group (*p*<0.05).

qRT-PCR showed that aggrecan and sox9 mRNA levels were highest at week 2 and decreased at week 3. Collagen II gene expression decreased over the 3 weeks. While the expression of collagen I increased during the 3 months in the blank and control groups, that in the cell group was not significantly different compared with the other two groups (Fig. 6A, B).

Western blotting analysis showed that the collagen II, aggrecan and sox9 levels were highest in the cell group and lowest in the blank group, while the collagen I level increased in all three groups and was highest in the blank group (Fig. 6C).

#### 3.4 Micro-CT assessment

**Figure 7:**
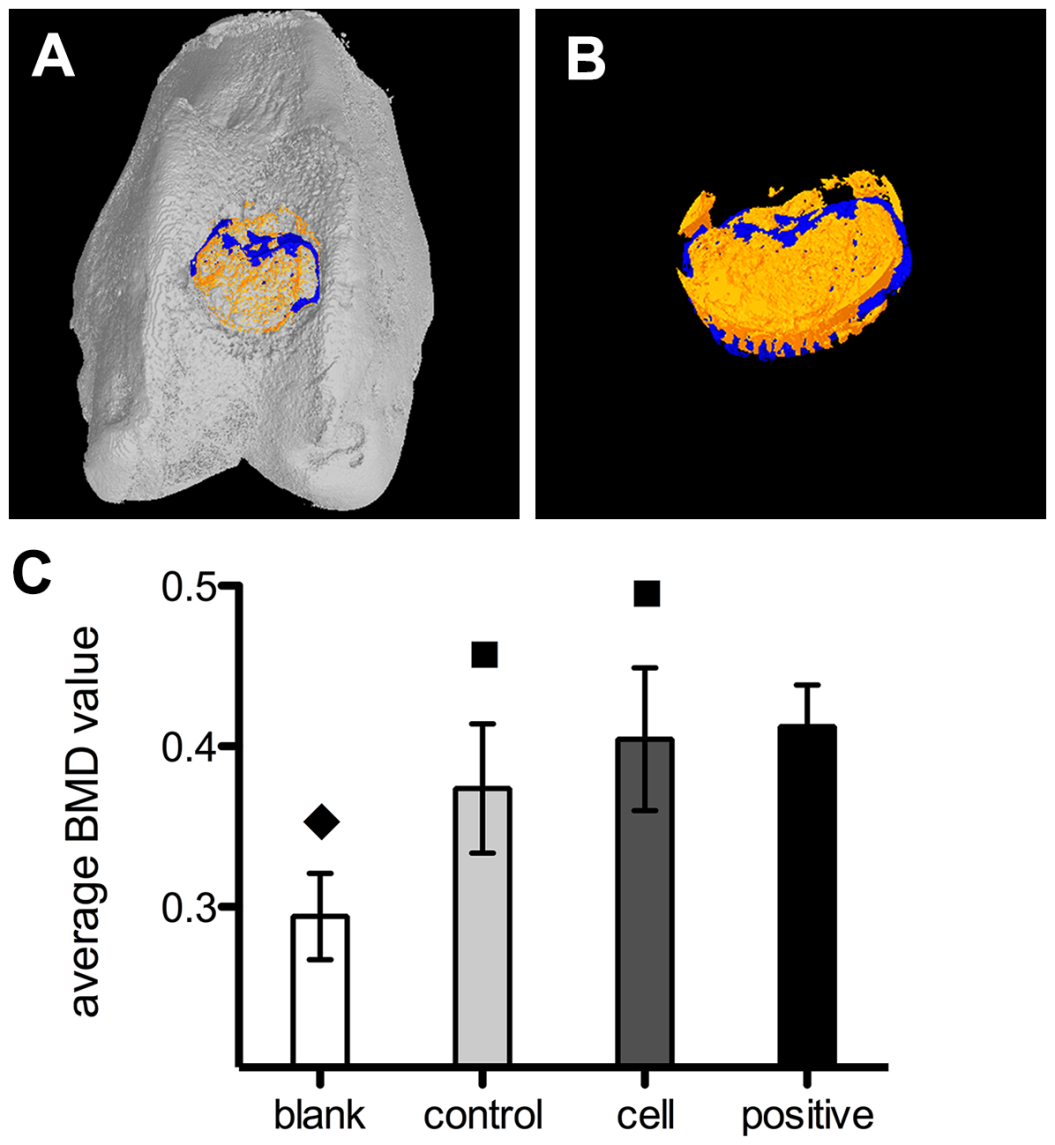
Assessment of subchondral bone restoration based on the micro-CT test. Bright yellow (**A, B**) represents regenerated bone and blue represents tissue of lower density; e.g., fibrous tissue. (**C**) Comparison of BMD among the three groups and normal bone tissue (positive group). ♦ Statistically significant difference compared with the positive group (*p*<0.01); ^▪^Statistically significant difference compared with the blank group (*p*<0.05).

Micro-CT was used to assess subchondral bone regeneration in the defect area. The calcified regenerated subchondral bone is shown in orange in the 3D images and tissue with lower density (such as fibrous tissue) is shown as dark blue. The average BMD was highest in the cell group (*p*<0.05). The average BMD values were not significantly different between the scaffold and control groups (Fig. 7).

## Discussion

In this study we investigated the effect of a 3D nanofibrous structure on the chondrogenic induction of iPSCs *in vitro* and the repair of an articular cartilage defect in a non-weight-bearing area. Our findings showed that the growth and chondrogenesis of iPSCs were enhanced by culture on a nanofibrous scaffold. Furthermore, when iPSC-seeded scaffolds were transplanted into the cartilage defect area, the restoration outcome was superior to that achieved by scaffold-only transplantation.

The current treatments for cartilage loss include autologous chondrocyte transplantation procedure, arthroscopic lavage and debridement, the subchondral bone microfracture technique, and osteochondral allograft [23]–[25]. These procedures may relieve the symptoms temporarily, but are often associated with problems such as donor site morbidity, loss of chondrocyte phenotype during *in vitro* expansion, fibrocartilage formation, and cartilage degeneration [26]–[28]. As stated previously, iPSCs are free of ethical issues because they are obtained from reprogrammed somatic cells, yet resemble ESCs in their multipotency and self-renewal capacity [7]. Diverse strategies have been employed to optimize the *in vivo* and *in vitro* chondrogenic induction of the two cell types, including direct differentiation *via* EB formation, high-density micromass induction, and co-culture with chondroprogenitor cells [29]–[31]. The high-density micromass method displayed a chondrogenic efficacy superior to that of direct plating of EBs, which is in agreement with the classic pellet method for chondrogenic induction of BMSCs or expansion of chondrocytes *in vitro* [29], [32]. This superiority is possibly due to the chondrogenic differentiation of ESCs being a context-dependent process and enhanced by 3D-culture systems, such as the pellet and high-density micromass systems [33]. Such 3D systems facilitate interactions between cells as well as between cells and the matrix, simulating the development of limb buds in which chondrogenesis is induced following condensation and consolidation of mesenchymal stem cells [34]. In this experiment, application of three drops of 15- µL medium containing 3×10^5^ cells on the scaffold created a micromass with sufficiently high cell density, resulting in generation of an appropriate chondrogenic microenvironment [35], [36].

An effective cell therapy for cartilage defects requires support from biomaterials or scaffolds. In the restoration of tissue defects, scaffolds can deliver cells or growth factors, provide a structure to which cells can attach and form tissue, and promote cell growth into the implant, both *in vitro* and *in vivo* [37]. These properties account for the superiority of scaffolds over plating cultures or 2D systems in terms of tissue structure restoration and function [34]. Furthermore, 3D systems with a fiber-deposited structure are superior to structures with a compress-molded feature or homogenous material [34]. Additionally, such scaffolds composed of randomly aligned fibers with evenly distributed diameters of hundreds of nanometers to several microns are readily fabricated by electrospinning methods. In the *in vitro* chondrogenic induction experiment the levels of chondrogenic markers were notably elevated in the scaffold group. This could be attributed to the unique properties of nanofibrous scaffolds; i.e., their high porosity and specialized surface area that degraded over 2 months. These features facilitate cartilage restoration as this is an ongoing process requiring an extended period of time.

Scaffold biocompatibility can also be affected by its components. Scaffolds composed of synthetic materials such as PLGA, PCL, or PLLA may have better mechanical properties but low biocompatibility. Alternatively, natural materials such as gelatin, collagen, or fibrin are more biocompatible but less mechanically supportive. A combination of the two types of material could create a bioscaffold with a balanced profile [38], [39]. SEM images showed cell attachment to the scaffold surface. Compared with cells in natural cartilaginous tissue, the cells cultured on a scaffold with nanofibrous structure displayed different morphological features characterized by protrusions stretching along the fibers. The chondrogenically induced iPSCs were attached to the scaffolds either in the form of clusters or were present individually separated by space, although seeded at a high density. A portion of the cells moved into the spaces between the fibers. These distinct cell features could be due to the nanofibrous structure and high porosity of the scaffold, and the incorporation of gelatin.

The CCK-8 test did not demonstrate scaffold superiority for cell proliferation compared with the control group. Immunofluorescence, PCR, and western blotting indicated that the cells on the scaffold produced higher levels of chondrogenic biomarkers than did cells on the plate at both the gene expression and protein levels. The distinction between the two groups could be attributed to the enhanced cell seeding and differentiation on the nanofibrous PCL/gelatin scaffold. In addition, the chondrogenic-inducing factor TGF-β3, a key promoter of chondrogenic stem cell differentiation, does not increase cell numbers [40], [41].

A full-thickness articular cartilage defect was created to examine the cartilage repair efficacy of the cell-scaffold complex *in vivo*. Two issues need to be addressed before iPSCs can be used to repair cartilage defects *in vivo*: immune rejection and teratoma formation. Reportedly, iPSCs-derived cells are not rejected after syngeneic transplantation [42]. In our study, the transplanted cells were xenogenic; therefore we used cyclosporin as an immunosuppressor to prolong the survival of the transplanted cells. Regarding teratoma formation, when undifferentiated mESCs were injected into rat osteochondral defects for repair, no teratomas were formed [43]. However, injection of mESCs into the knee joint cavity resulted in formation of teratomas that destroyed the entire joint [44]. In our study, as a precaution, iPSCs were cultured on the scaffolds and maintained in the defect area. Healing of full-thickness cartilage defects is difficult using spontaneously generated fibrous cartilage [45], which explains the fissures and exposure of subchondral bone in the defect area of the blank group. The defects covered only with the scaffold were repaired better than in the control group, but the regenerated tissue in the defect area was essentially fibrocartilaginous, as indicated by the weak safranin-O staining of the regenerated tissue compared with the normal area. Immunohistochemical staining of collagen type II was also attenuated while that of collagen type I was intensified. H&E staining showed chondrocytes dispersed sparsely in the regenerated tissue. The cell group showed better repair, as indicated by a greater number of chondrocytes and upregulated expression of collagen II and aggrecan. The PCR and western blot analyses suggested that, the gene expressions of aggrecan and sox9 were highest at the second week, and decreased at the third week, while the production of these two cartilaginous markers were kept increasing throughout the three weeks. The gene expression of col-II was decreasing during the three weeks, while its production kept increasing, as was shown by the western blotting analysis. This is a result of a discrepancy between transcription and translation inside the cells. During regeneration of cartilaginous tissue, cartilage ECM production increases continuously to form the regenerated tissue. However, during tissue regeneration, gene expression is initially upregulated until a certain level is reached. Then, expression may be maintained or decreased slightly. The enhancement of ECM synthesis was likely attributable to the use of the cell–scaffold combination. The nanofibrous scaffolds covered the defect area and not only provided the implanted cells with mechanical and spatial support but also a microenvironment similar to that of normal cartilage, facilitating the exchange of nutrients and waste, which promoted iPSC differentiation and bioactivity.

## Conclusions

Our findings suggested the PCL/gelatin scaffold was biocompatible with both the iPSCs and articular cartilage. In addition, the scaffold enhanced the chondrogenesis of iPSCs due to its nanofibrous structure and hydrophilicity, which was due to the addition of gelatin. Use of the cell–scaffold combination showed evidence of articular cartilage defect repair, resulting in an enhanced gross appearance and histological improvements, higher cartilage-specific gene expression and protein levels as well as subchondral bone regeneration, suggesting a possible application in the treatment of articular cartilage defects.
